# Supplementary material for: Epigenetic Silencing of HER2 Expression during Epithelial-Mesenchymal Transition Leads to Trastuzumab Resistance in Breast Cancer
Source: Life (Basel). 2021 Aug 24;11(9):868. doi: 10.3390/life11090868 (PMC8472246; doi:10.3390/life11090868)
Supplement: Supplementary file 1 [file life-11-00868-s001.zip › life-1333507-supplementary.pdf]

Supplementary Materials

Table S1. GEO series and samples accession IDs of expression and methylation arrays data analyzed in this thesis.

GEO Series	GEO Samples	Data Type	Reference
GSE110189	GSM2981936	Untreated CHO-K1 cells	[1]
	GSM2981937	CHO-K1 cells treated with trastuzumab+pertuzumab	
	GSM2981938	Untreated CHO-K6	
	GSM2981939	CHO-K6 treated with trastuzumab	
	GSM2981940	CHO-K6 treated with pertuzumab	
	GSM2981941	CHO-K6 treated with trastuzumab+ pertuzumab	
GSE50811	GSM1229992, GSM1229993, GSM1229994, GSM1230001, GSM1230002, GSM1230003, GSM1230010, GSM1230011, GSM1230012, GSM1230019, GSM1230020, GSM1230021, GSM1230028, GSM1230029, GSM1230030, GSM1230037, GSM1230038, GSM1230045, GSM1230046, GSM1230047, GSM1230054, GSM1230055, GSM1230056, GSM1230063, GSM1230064, GSM1230065, GSM1230072, GSM1230073, GSM1230074, GSM1230081, GSM1230082, GSM1230083, GSM1230090, GSM1230091, GSM1230092, GSM1230099, GSM1230100, GSM1230101, GSM1230108, GSM1230109, GSM12301, GSM1230117, GSM1230118, GSM123011910, GSM1230126, GSM1230127, GSM1230134, GSM1230135, GSM1230136, GSM1230143, GSM1230144, GSM1230145, GSM1230152, GSM1230153, GSM1230154, GSM1230161, GSM1230162, GSM1230163, GSM1230170, GSM1230171, GSM1230172,	Expression profiling of breast cancer cell lines by array	[2]

	GSM1230179, GSM1230180, GSM1230181, GSM1230188, GSM1230189, GSM1230190, GSM1230197, GSM1230198, GSM1230199, GSM1230206, GSM1230207, GSM1230208, GSM1230215, GSM1230216, GSM1230217, GSM1230224, GSM1230225, GSM1230226		
GSE44838	GSM1092241, GSM1092242, GSM1092251, GSM1092253, GSM1092255, GSM1092259, GSM1092260, GSM1092261, GSM1092263, GSM1092264,	Expression profiling of breast cancer cell lines by array	[3]
	GSM1092267, GSM1092268, GSM1092277, GSM1092279, GSM1092281, GSM1092285, GSM1092286, GSM1092287, GSM1092289, GSM1092290	Methylation profiling of breast cancer cell lines by genome tiling array	
GSE16179	GSM799168, GSM799169, GSM799170	Lapatinib-sensitive BT474 cell lines	[4]
	GSM799174, GSM799175, GSM799176	Lapatinib-resistant BT474 cell lines	
GSE17708	GSM442026, GSM442027, GSM442028	Untreated A549	[5]
	GSM442029, GSM442030, GSM442031	A549 treated with TGFβ1 for 0.5 hour	
	GSM442032, GSM442033, GSM442034	A549 treated with TGFβ1 for 1 hour	
	GSM442035, GSM442036	A549 treated with TGFβ1 for 2 hours	
	GSM442037, GSM442038, GSM442039	A549 treated with TGFβ1 for 4 hours	
	GSM442040, GSM442041, GSM442042	A549 treated with TGFβ1 for 8 hours	
	GSM442043, GSM442044, GSM442045	A549 treated with TGFβ1 for 16 hours	
	GSM442046, GSM442047, GSM442048	A549 treated with TGFβ1 for 24 hours	

GSM442049, GSM442050, GSM442051	A549 treated with TGFβ1 for 72 hours

Table S2. Cistrome DB and GEO accession IDs of ChIP-seq data analyzed in this thesis.

Factor	X-seq	Cell line	Cistrome DB ID	GEO sample ID	Reference
Tn5	ATAC-seq	MCF7	79676	GSM2714245	[6]
Tn5	ATAC-seq	MDA-MB-231	65718	GSM2439559	[7]
DNase	DNase-seq	MCF7	40995	GSM1008581	[8]
DNase	DNase-seq	MDA-MB-231	78267	GSM2242137	[9]
FOXA1	ChIP-seq	MDA-MB-453	36842	GSM1099031	[10]
FOXA1	ChIP-seq	MCF7	2320	GSM659787	[11]
E2F1	ChIP-seq	MCF7	2281	GSM699986	[12]
E2F1	ChIP-seq	MDA-MB-231	75043	GSM2501567	[9]
H2BK120ub	ChIP-seq	HCC-1954	85603	GSM2258929	[13]
H2BK120ub	ChIP-seq	SKBR3	82286	GSM2258950	[13]
H2BK120ub	ChIP-seq	AU565	85597	GSM2258923	[13]
H2BK120ub	ChIP-seq	MDA-MB-361	81479	GSM2258935	[13]
H2BK120ub	ChIP-seq	MCF7	86405	GSM2258947	[13]
H2BK120ub	ChIP-seq	MDA-MB-231	81472	GSM2258932	[13]
H2BK120ub	ChIP-seq	MDA-MB-468	86411	GSM2258941	[13]
H3K39me3	ChIP-seq	HCC-1954	88403	GSM2258835	[13]
H3K39me3	ChIP-seq	SKBR3	87169	GSM2258798	[13]
H3K39me3	ChIP-seq	AU565	88152	GSM2258816	[13]
H3K39me3	ChIP-seq	MDA-MB-361	81935	GSM2258762	[13]
H3K39me3	ChIP-seq	MCF7	74303	GSM2483406	[13]
H3K39me3	ChIP-seq	MDA-MB-231	2408	GSM425485	[14]
H3K39me3	ChIP-seq	MDA-MB-468	82822	GSM2258889	[13]
K3K79me2	ChIP-seq	HCC-1954	85976	GSM2258841	[13]
K3K79me2	ChIP-seq	SKBR3	84023	GSM2258805	[13]
K3K79me2	ChIP-seq	AU565	84313	GSM2258823	[13]
K3K79me2	ChIP-seq	MDA-MB-361	81942	GSM2258769	[13]
K3K79me2	ChIP-seq	MCF7	86291	GSM2258733	[13]
K3K79me2	ChIP-seq	MDA-MB-231	88654	GSM2258858	[13]
K3K79me2	ChIP-seq	MDA-MB-468	87026	GSM2258894	[13]
H3K4me1	ChIP-seq	HCC-1954	88402	GSM2258836	[13]
H3K4me1	ChIP-seq	SKBR3	84019	GSM2258801	[13]
H3K4me1	ChIP-seq	AU565	88154	GSM2258818	[13]
H3K4me1	ChIP-seq	MDA-MB-361	81941	GSM2258764	[13]
H3K4me1	ChIP-seq	MCF7	82432	GSM2258728	[13]
H3K4me1	ChIP-seq	MDA-MB-231	68383	GSM2036932	[13]
H3K4me1	ChIP-seq	MDA-MB-468	87022	GSM2258890	[13]
H3K4me3	ChIP-seq	HCC-1954	8399	GSM721134	[15]
H3K4me3	ChIP-seq	SKBR3	82540	GSM2258803	[13]
H3K4me3	ChIP-seq	AU565	84311	GSM2258821	[13]
H3K4me3	ChIP-seq	MDA-MB-361	81939	GSM2258766	[13]
H3K4me3	ChIP-seq	MCF7	86289	GSM2258731	[13]
H3K4me3	ChIP-seq	MDA-MB-231	68122	GSM1700393	[16]

H3K4me3	ChIP-seq	MDA-MB-468	54526	GSM1429760	[17]
H3K9ac	ChIP-seq	HCC-1954	84601	GSM2258842	[13]
H3K9ac	ChIP-seq	SKBR3	84020	GSM2258806	[13]
H3K9ac	ChIP-seq	AU565	84254	GSM2258824	[13]
H3K9ac	ChIP-seq	MCF7	86292	GSM2258734	[13]
H3K9ac	ChIP-seq	MDA-MB-231	58110	GSM1619768	[18]
H3K9ac	ChIP-seq	MDA-MB-468	87027	GSM2258897	[13]
H3K27ac	ChIP-seq	HCC-1954	88400	GSM2258830	[13]
H3K27ac	ChIP-seq	SKBR3	87173	GSM2258794	[13]
H3K27ac	ChIP-seq	MDA-MB-361	85478	GSM2258758	[13]
H3K27ac	ChIP-seq	MCF7	82438	GSM2258722	[13]
H3K27ac	ChIP-seq	MDA-MB-231	61914	GSM1855992	[19]
H3K27ac	ChIP-seq	MDA-MB-468	82828	GSM2258884	[13]
H4K8ac	ChIP-seq	HCC-1954	81475	GSM2258931	[13]
H4K8ac	ChIP-seq	SKBR3	82288	GSM2258952	[13]
H4K8ac	ChIP-seq	AU565	85599	GSM2258925	[13]
H4K8ac	ChIP-seq	MDA-MB-361	81477	GSM2258937	[13]
H4K8ac	ChIP-seq	MCF7	86413	GSM2258949	[13]
H4K8ac	ChIP-seq	MDA-MB-231	81478	GSM2258934	[13]
H4K8ac	ChIP-seq	MDA-MB-468	86409	GSM2258943	[13]
H3K9me3	ChIP-seq	HCC-1954	84600	GSM2258845	[13]
H3K9me3	ChIP-seq	SKBR3	84024	GSM2258808	[13]
H3K9me3	ChIP-seq	AU565	84308	GSM2258826	[13]
H3K9me3	ChIP-seq	MDA-MB-361	84978	GSM2258772	[13]
H3K9me3	ChIP-seq	MCF7	86294	GSM2258736	[13]
H3K9me2	ChIP-seq	MDA-MB-231	58111	GSM1619769	[18]
H3K9me3	ChIP-seq	MDA-MB-468	87019	GSM2258898	[13]
H3K27me3	ChIP-seq	HCC-1954	88398	GSM2258832	[13]
H3K27me3	ChIP-seq	SKBR3	87171	GSM2258796	[13]
H3K27me3	ChIP-seq	AU565	88150	GSM2258814	[13]
H3K27me3	ChIP-seq	MDA-MB-361	81936	GSM2258761	[13]
H3K27me3	ChIP-seq	MCF7	82436	GSM2258724	[13]
H3K27me3	ChIP-seq	MDA-MB-231	88660	GSM2258850	[13]
H3K27me3	ChIP-seq	MDA-MB-468	82827	GSM2258887	[13]

References

1. Nami, B.; Maadi, H.; Wang, Z. The Effects of Pertuzumab and Its Combination with Trastuzumab on HER2 Homodimerization and Phosphorylation. *Cancers (Basel).* **2019**, *11*.
2. Dezso, Z.; Oestreicher, J.; Weaver, A.; Santiago, S.; Agoulnik, S.; Chow, J.; Oda, Y.; Funahashi, Y. Gene expression profiling reveals epithelial mesenchymal transition (EMT) genes can selectively differentiate eribulin sensitive breast cancer cells. *PLoS ONE* **2014**, *9*, e106131.
3. Di Cello, F.; Cope, L.; Li, H.; Jeschke, J.; Wang, W.; Baylin, S.B.; Zahnow, C.A. Methylation of the claudin 1 promoter is associated with loss of expression in estrogen receptor positive breast cancer. *PLoS ONE* **2013**, *8*, e68630.
4. Liu, L.; Greger, J.; Shi, H.; Liu, Y.; Greshock, J.; Annan, R.; Halsey, W.; Sathe, G.M.; Martin, A.-M.; Gilmer, T.M. Novel mechanism of lapatinib resistance in HER2-positive breast tumor cells: Activation of AXL. *Cancer Res.* **2009**, *69*, 6871–6878.
5. Sartor, M.A.; Mahavisno, V.; Keshamouni, V.G.; Cavalcoli, J.; Wright, Z.; Karnovsky, A.; Kuick, R.; Jagadish, H. V; Mirel, B.; Weymouth, T.; et al. ConceptGen: A gene set enrichment and gene set relation mapping tool. *Bioinformatics* **2010**, *26*, 456–463.
6. Porter, J.R.; Fisher, B.E.; Baranello, L.; Liu, J.C.; Kambach, D.M.; Nie, Z.; Koh, W.S.; Luo, J.; Stommel, J.M.; Levens, D.; et al. Global Inhibition with Specific Activation: How p53 and MYC Redistribute the Transcriptome in the DNA Double-Strand Break Response. *Mol. Cell* **2017**, *67*, 1013–1025.e9.

7. Bustos, M.A.; Salomon, M.P.; Nelson, N.; Hsu, S.C.; DiNome, M.L.; Hoon, D.S.B.; Marzese, D.M. Genome-wide chromatin accessibility, DNA methylation and gene expression analysis of histone deacetylase inhibition in triple-negative breast cancer. *Genomics data* **2017**, *12*, 14–16.
8. Thurman, R.E.; Rynes, E.; Humbert, R.; Vierstra, J.; Maurano, M.T.; Haugen, E.; Sheffield, N.C.; Stergachis, A.B.; Wang, H.; Vernot, B.; et al. The accessible chromatin landscape of the human genome. *Nature* **2012**, *489*, 75–82.
9. Gallenne, T.; Ross, K.N.; Visser, N.L.; Salony; Desmet, C.J.; Wittner, B.S.; Wessels, L.F.A.; Ramaswamy, S.; Peeper, D.S. Systematic functional perturbations uncover a prognostic genetic network driving human breast cancer. *Oncotarget* **2017**, *8*, 20572–20587.
10. Ni, M.; Chen, Y.; Fei, T.; Li, D.; Lim, E.; Liu, X.S.; Brown, M. Amplitude modulation of androgen signaling by c-MYC. *Genes Dev.* **2013**, *27*, 734–748.
11. Joseph, R.; Orlov, Y.L.; Huss, M.; Sun, W.; Kong, S.L.; Ukil, L.; Pan, Y.F.; Li, G.; Lim, M.; Thomsen, J.S.; et al. Integrative model of genomic factors for determining binding site selection by estrogen receptor-alpha. *Mol. Syst. Biol.* **2010**, *6*, 456.
12. Cao, A.R.; Rabinovich, R.; Xu, Mc.; Xu, X.; Jin, V.X.; Farnham, P.J. Genome-wide analysis of transcription factor E2F1 mutant proteins reveals that N- and C-terminal protein interaction domains do not participate in targeting E2F1 to the human genome. *J. Biol. Chem.* **2011**, *286*, 11985–11996.
13. Franco, H.L.; Nagari, A.; Malladi, V.S.; Li, W.; Xi, Y.; Richardson, D.; Allton, K.L.; Tanaka, K.; Li, J.; Murakami, S.; et al. Enhancer transcription reveals subtype-specific gene expression programs controlling breast cancer pathogenesis. *Genome Res.* **2018**, *28*, 159–170.
14. Hodges, E.; Smith, A.D.; Kendall, J.; Xuan, Z.; Ravi, K.; Rooks, M.; Zhang, M.Q.; Ye, K.; Bhattacharjee, A.; Brizuela, L.; et al. High definition profiling of mammalian DNA methylation by array capture and single molecule bisulfite sequencing. *Genome Res.* **2009**, *19*, 1593–1605.
15. Hon, G.C.; Hawkins, R.D.; Caballero, O.L.; Lo, C.; Lister, R.; Pelizzola, M.; Valsesia, A.; Ye, Z.; Kuan, S.; Edsall, L.E.; et al. Global DNA hypomethylation coupled to repressive chromatin domain formation and gene silencing in breast cancer. *Genome Res.* **2012**, *22*, 246–258.
16. Messier, T.L.; Gordon, J.A.R.; Boyd, J.R.; Tye, C.E.; Browne, G.; Stein, J.L.; Lian, J.B.; Stein, G.S. Histone H3 lysine 4 acetylation and methylation dynamics define breast cancer subtypes. *Oncotarget* **2016**, *7*, 5094–5109.
17. Zhu, J.; Sammons, M.A.; Donahue, G.; Dou, Z.; Vedadi, M.; Getlik, M.; Barsyte-Lovejoy, D.; Al-awar, R.; Katona, B.W.; Shilatifard, A.; et al. Gain-of-function p53 mutants co-opt chromatin pathways to drive cancer growth. *Nature* **2015**, *525*, 206–211.
18. Vasudevan, D.; Hickok, J.R.; Bovee, R.C.; Pham, V.; Mantell, L.L.; Bahroos, N.; Kanabar, P.; Cao, X.-J.; Maienschein-Cline, M.; Garcia, B.A.; et al. Nitric Oxide Regulates Gene Expression in Cancers by Controlling Histone Posttranslational Modifications. *Cancer Res.* **2015**, *75*, 5299–5308.
19. Takaku, M.; Grimm, S.A.; Shimbo, T.; Perera, L.; Menafra, R.; Stunnenberg, H.G.; Archer, T.K.; Machida, S.; Kurumizaka, H.; Wade, P.A. GATA3-dependent cellular reprogramming requires activation-domain dependent recruitment of a chromatin remodeler. *Genome Biol.* **2016**, *17*, 36.
